# Dynamic Changes of Brain Activity in Different Responsive Groups of Patients with Prolonged Disorders of Consciousness

**DOI:** 10.3390/brainsci13010005

**Published:** 2022-12-20

**Authors:** Chen Chen, Jinying Han, Shuang Zheng, Xintong Zhang, Haibo Sun, Ting Zhou, Shunyin Hu, Xiaoxiang Yan, Changqing Wang, Kai Wang, Yajuan Hu

**Affiliations:** 1 Department of Neurology, The First Affiliated Hospital of Anhui Medical University, Hefei 230032, China; 2 Collaborative Innovation Center of Neuropsychiatric Disorders and Mental Health, Hefei 230032, China; 3 Anhui Province Key Laboratory of Cognition and Neuropsychiatric Disorders, Hefei 230032, China; 4 The School of Mental Health and Psychological Sciences, Anhui Medical University, Hefei 230032, China; 5 The First Clinical College of Anhui Medical University, Hefei 230032, China; 6 Department of Neurology, The Second Affiliated Hospital of Anhui University of Chinese Medicine, Hefei 230001, China; 7 Department of Neurorehabilitation, Hefei Anhua Trauma Rehabilitation Hospital, Hefei 230011, China; 8 Hefei Comprehensive National Science Center, Institute of Artificial Intelligence, Hefei 230032, China

**Keywords:** disorders of consciousness (DOCs), Coma Recovery Scale-Revised (CRS-R), electroencephalogram microstate, high-definition transcranial direct current stimulation (HD-tDCS)

## Abstract

As medical technology continues to improve, many patients diagnosed with brain injury survive after treatments but are still in a coma. Further, multiple clinical studies have demonstrated recovery of consciousness after transcranial direct current stimulation. To identify possible neurophysiological mechanisms underlying disorders of consciousness (DOCs) improvement, we examined the changes in multiple resting-state EEG microstate parameters after high-definition transcranial direct current stimulation (HD-tDCS). Because the left dorsolateral prefrontal cortex is closely related to consciousness, it is often chosen as a stimulation target for tDCS treatment of DOCs. A total of 21 patients diagnosed with prolonged DOCs were included in this study, and EEG microstate analysis of resting state EEG datasets was performed on all patients before and after interventions. Each of them underwent 10 anodal tDCS sessions of the left dorsolateral prefrontal cortex over 5 consecutive working days. According to whether the clinical manifestations improved, DOCs patients were divided into the responsive (RE) group and the non-responsive (N-RE) group. The dynamic changes of resting state EEG microstate parameters were also analyzed. After multiple HD-tDCS interventions, the duration and coverage of class C microstates in the RE group were significantly increased. This study also found that the transition between microstates A and C increased, while the transition between microstates B and D decreased in the responsive group. However, these changes in EEG microstate parameters in the N-RE group have not been reported. Our findings suggest that EEG neural signatures have the potential to assess consciousness states and that improvement in the dynamics of brain activity was associated with the recovery of DOCs. This study extends our understanding of the neural mechanism of DOCs patients in consciousness recovery.

## 1. Introduction

In recent years, more and more patients with severe cerebral injuries continue to fall into a coma after aggressive treatments in the clinic. After the acute phase of coma, some patients regain consciousness, but there are still many patients with disorders of consciousness (DOCs). According to temporal criteria, acute DOCs present for less than 28 days, while prolonged DOCs indicate that the brain injury occurred at least 28 days in the past [1]. The most common causes of DOCs are cardiac arrest, traumatic brain injury, cerebral hemorrhage, and ischemic stroke. DOCs can be further divided into unresponsive wakefulness syndrome (known as vegetative states, VS), minimally conscious states (MCS) [2], and cognitive movement dissociation [3]. Minimally conscious states can be further divided into MCS− and MCS+. Patients in MCS– demonstrate visual pursuit and/or fixation, oriented movements, and localization to pain [4]. While in MCS+, patients may follow commands and exhibit intelligible verbalization and/or intentional communication [5]. Patients with MCS are considered to emerge from minimally conscious states (EMCS) when they demonstrate functional communication or the ability to use two different objects [6]. Self-recovery from DOCs is a lengthy and unpredictable process, placing a burden on the healthcare system and the patient’s family, so effective intervention for patients with DOCs is critical.

Transcranial direct current stimulation (tDCS) is a non-invasive method for modulating cortical excitability that may hold great therapeutic potential by inducing regional neuroplastic changes after brain injury. In 2009, a new electrode configuration called high-definition tDCS (HD-tDCS) was designed to increase the focus of stimulation [7], allowing for more accurate targeting of specific brain regions [8]. The left dorsolateral prefrontal cortex (DLPFC) receives inputs encoding vision, motion, spatial orientation, and tactile sensation from multimodal association cortices and projects to various subcortical monoaminergic and cholinergic nuclei involved in arousal and attention [9]. It was found that tDCS over the left DLPFC improves the clinical condition of some DOCs patients [10].

The highly dynamic waking cortical electroencephalogram (EEG) pattern is interspersed with brief periods in which activity remains semi-stable [11]. These transient periods of stability have different topographical representations, termed microstates, that reflect changes in large-scale neural network activity. Microstates become shorter and more frequent with age and show a change in the total time distribution covered by each microstate [12]. Recent evidence suggests that resting-state activity occurs coherently in a large number of neural populations, indicating that entire brain networks are continuously active in resting states. Resting-state functional connectivity studies also revealed the rich complexity of resting-state activity, which is characterized by the temporal correlation of neuronal activity in different regions. Brain regions that exhibit functional connectivity are thought to be organized into discrete networks related to different functions, including many resting-state networks (RSNs). Among these is a default mode network (DMN), which is active in the negative state of a task but becomes inactive for a large number of cognitive tasks [13]. The link between EEG microstates and RSNs was identified by functional magnetic resonance imaging (fMRI), indicating that RSNs of fMRI may be the RSNs that produce the microstates [14]. These studies indicated that the brain is organized into multiple overlapping large-scale functional networks [15]. It was reported that microstate A, which is believed to reflect the activity of an auditory or sensorimotor network, was associated with a negative blood oxygen level-dependent signal in the superior and middle temporoparietal cortices. Subsequent studies revealed that microstate class B, viewed as reflecting visual network activity, was associated with negative BOLD activation in the striate, extrastriate, and occipital cortex, while microstate class C, widely believed to reflect the activity of a salience network, was mainly associated with BOLD-positive activation in the inferior frontal cortex, dorsal anterior cingulate cortex, and right insular regions, and microstate class D, thought to reflect the activity of an attentional network, with negative BOLD activation in the right ventral and dorsal regions of the frontal and parietal cortex [16].

EEG microstate analysis has been used to investigate the spatial and temporal abnormalities of whole-brain neural networks [17] in diseases such as schizophrenia [18], dementia [19], epilepsy [20], and narcolepsy [21], as well as to characterize different states of consciousness, including sleep, hypnosis, and meditation. One study pointed out that four main microstates persist throughout all stages of non-rapid eye movement sleep. In N1 and N3 of non-rapid eye movement sleep, the microstate maps and features were close to wakefulness [22]. In addition, the microstates of healthy people during initial rest, mild hypnosis, deep hypnosis, and final recovery were analyzed, and the four reported microstate levels were very similar to the standard four microstate levels A, B, C, and D [23]. One study used EEG and fMRI to compare the spatial range and temporal dynamics of DMN during meditation. They reported that the duration and frequency of occurrence of the DMN-microstates are useful metrics of meditation-induced altered consciousness and increase systematically during rest and during meditation [24].

In recent years, more and more studies have focused on the changes in EEG microstates under different conditions of consciousness. Previous studies have fully explored the neuromechanism of consciousness and unconsciousness using EEG analysis [1,25]. Fingelkurts et al. reported that altered states of consciousness were associated with a decreased number of EEG microstate types, and unconsciousness was related to the lack of diversity in EEG alpha-rhythmic microstates. What is more, the probability of the occurrence and duration of delta (1–2.5 Hz), theta (3–7 Hz), and slow-alpha (7.5–8.5 Hz) rhythmic microstates was associated with unconsciousness, while the probability of the occurrence and duration of fast-alpha (9–13 Hz) rhythmic microstates was associated with consciousness [26]. They also indicated that delta, theta, and slow-alpha oscillations were less common in MCS patients rather than in VS patients, and only fast-alpha oscillations only can be found in MCS patients [27]. In addition, Stefan et al. pointed out that the percentage of time in microstate D in the alpha (8–13 Hz) band was the best index for the classification of VS and MCS [28]. Recent microstate studies of DOC patients have shown that seven cortical activation microstates with distinct spatial distributions were identified, and there were significant differences between the MCS and VS patients in microstate properties, such as spatial activation patterns, temporal dynamics, state transitions, and connection construction [29]. Additionally, a study of consciousness indexing and prognostic prediction in DOCs patients noted that the percentage of time spent in microstate D in the alpha frequency band performed best at distinguishing MCS from UWS patients [28]. Another study on fMRI showed a reduction in metastable and functional network repertoire in patients with UWS compared to those with MCS. The residence time of the DMN and subcortical frontotemporal parietal network was reduced, and non-stationality was lost in UWS patients compared with MCS patients [30]. All these studies provide new perspectives and important information for the mechanism of consciousness disorder.

However, there are few studies on resting-state EEG microstates related to arousal-promoting of DOCs. By detecting the changes in brain microstates after HD-tDCS interventions, this study provides an important perspective for understanding the brain of DOCs patients and offers more useful evidence of the neural mechanisms involved in consciousness recovery.

## 2. Materials and Methods

### 2.1. Participants

From September 2020 to December 2021, DOCs patients from the Department of Neurology of the First Affiliated Hospital of Anhui Medical University (Hefei, China), the Department of Neurology of the Second Affiliated Hospital of Anhui University of Chinese Medicine (Hefei, China), and the Department of Neurorehabilitation of Hefei Anhua Trauma Rehabilitation Hospital (Hefei, China) were included in this study. Inclusion criteria include (1) patients diagnosed as VS/MCS based on CRS-R assessment by at least two professional neurologists (if there is disagreement on the score, another professional neurologist decides), (2) the age requirement is between 18 and 75, (3) even if the patient is admitted to the ICU, vital signs need to be stable, (4) no neuromuscular blockers and sedatives have been used within 24 h before the study and the entire protocol, (5) no improvement in the state of consciousness was observed within one week before the start of the study. The exclusion criteria were (1) previous or current diagnosis of severe neurocognitive degenerative diseases, (2) metal implantation in the head, (3) previous surgical procedures resulted in skull defect, (4) previous history of epilepsy, (5) patients with frontal lobe lesions, (6) previous received invasive or non-invasive neuroregulatory interventions that may have caused changes in brain function.

All rehabilitation protocols and medication regimens were maintained as usual during the study. Written informed consent was obtained from the patients’ caregivers, and the study was approved by the ethics committee of the First Affiliated Hospital of Anhui Medical University (Hefei, China).

### 2.2. Assessment of Coma Recovery Scale

State of consciousness was assessed by two trained physicians in three different time periods using the Coma Recovery Scale-Revised (CRS-R), which has demonstrated good reliability and validity for the behavioral evaluation of DOCs patients [31]. Total score ranges from zero to twenty-three, with higher scores indicating better neurological function and prognosis. Patients were also divided into responsive and non-responsive groups (RE and N-RE, respectively) according to whether the CRS-R score was improved after HD-tDCS interventions.

### 2.3. Stimulation Protocol

**Figure 1:**
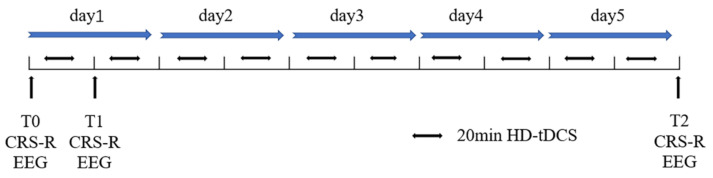
Stimulation Protocol. T0, time before HD-tDCS; T1, after a single session of HD-tDCS; T2, after 10 sessions of HD-tDCS for 5 days. All DOCs patients received HD-tDCS 10 times (2 mA, 20 min) for 5 consecutive days over the left dorsolateral prefrontal cortex by the same operator.

Stimulation was performed using a direct current stimulator (Neuroelectrics, Barcelona, Spain) linked to one anodal electrode and four cathodal electrodes. The anodal electrode was placed over the left dorsolateral prefrontal cortex (DLPFC; F3 in the 10–20 international system of EEG placement), and the four cathodal electrodes (AFz, FCz, F7, and C5 in the 10–20 international system EEG placement) were placed around the anodal electrode to form a current loop. The current was ramped up to 2 mA over 30 s before the start of each session. The current was maintained at 2 mA for 20 min and then slowly ramped down over 30 s [32,33]. All patients received 10 HD-tDCS sessions for 5 consecutive days, and the patients’ CRS-R scores and EEG were recorded before and after the treatment, as shown in Figure 1.

### 2.4. EEG Data Acquisition and Pre-Processing

Patient EEGs were recorded for at least 6 min from 19 channels (O1, O2, P3, P4, Pz, T5, T6, C3, C4, Cz, T3, T4, F3, F4, Fz, F7, F8, Fp1, Fp2) using Ag/AgCl pin electrodes connected to a polygraph amplifier (EEG-1200C, Nihon Kohden, Japan). The sampling rate was 200 Hz, and skin/electrode impedance was maintained below 5 kΩ at all electrode sites. Pre-processing was conducted using EEGLAB software version 13.0b running in the MATLAB environment (Version 2013b, MathWorks Inc.; Natick, MA, USA). The 50 Hz power signal was removed from the raw EEG single using a notch filter. The EEG signal was then band-pass filtered between 0.1 and 40 Hz [34]. Independent component analysis was used to identify and remove artifact-relevant components, including eye movements and muscle activation [35]. The selected artifact-free epochs were average referenced and rejected when greater than ±150 uV [36].

### 2.5. Microstate Analysis

Microstates were extracted from each patient’s EEG recordings using the Microstate plug-in for EEGLAB Toolbox [37]. Briefly, the EEG recordings of all DOCs patients were first aggregated into one dataset to derive microstate prototypes, which were then back-fitted to each individual EEG record. After defining EEG microstates for each patient, statistical time parameters were calculated.

### 2.6. Microstate Segmentation

Microstate prototypes were calculated from the aggregated EEG dataset of all patients. First, the spatial standard deviation of the EEG signal across all channels (global field power (GFP)) was calculated. Then, GFP sequences were grouped according to topographical similarity using a modified version of the K-mean clustering algorithm [37]. This analysis yielded four microstate class topographies (A, B, C, and D), a number previously deemed optimal and permitting comparison with the existing literature [17].

### 2.7. Microstate Parameters

The microstate parameters global explained variance (GEV), state single-episode duration, state occurrence frequency, relative proportion of time in a particular state (coverage), and transition dynamics (syntax) were then calculated as described [37]. GEV is a measure of how similar each EEG sample is to the assigned microstate prototype [38]. The GEV criterion theoretically (and most of the time effectively) becomes monotonically larger when increasing the number of clusters. Duration was defined as the average length of time in the same microstate class (i.e., the time without change in microstate), occurrence as the average number of events for each class per second, and coverage as the proportion (%) of total time in each microstate class. Finally, syntax refers to the rules governing the concatenation of microstates [39]. For each participant, the number of transitions from one class to any other class was counted and then normalized to all between-class transitions.

### 2.8. Statistical Analysis

Demographic and resting-state EEG microstate data were compared between RE and N-RE groups using SPSS 19.0 (IBM, Armonk, NY, USA). Continuous variables are expressed as mean±standard deviation and categorical variables as a percentage (%). Categorical variables were compared using the χ^2^ test and continuous variables by *t*-test if normally distributed or the Mann–Whitney U test if non-normally distributed. Microstate class transitions after stimulation were compared to baseline by Student’s *t*-test with FDR correction for multiple comparisons. All other microstate class parameters measured after stimulation were compared to corresponding baseline values by repeated measures ANOVA. *p* < 0.05 was considered statistically significant for all tests.

## 3. Results

### 3.1. Demographic and Clinical Behavioral Results

Twenty-one patients with prolonged DOCs (fifteen males and six females) due to trauma (four patients), hypoxic-ischemic encephalopathy (HIE) (four patients), cerebral hemorrhage (eight patients), cerebral infarction (four patients), or disseminated encephalomyelitis (one patient) were included in this study. Twelve patients (three females and nine males) demonstrated improved (higher) CRS-R scores following stimulation and were defined as responsive (RE group), while the remaining nine patients (three females and six males) demonstrated unchanged or lower CRS-R scores (N-RE group). There was no group difference (RE vs. N-RE) in mean age ((59.00 ± 9.95) years vs. (50.33 ± 15.80) years, t = −1.542, *p* = 0.140), mean duration since onset ((85.25 ± 83.52) days vs. (81.88 ± 57.59) days, t = −0.103, *p* = 0.919), sex ratio (z = −0.408, *p* = 0.754), or etiology (χ^2^ = 0.889, *p* = 0.346). Table 1 summarizes the demographic and clinical features of all study patients.

**Figure 2:**
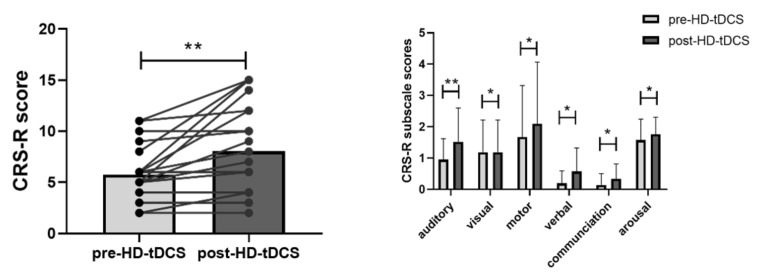
Changes in total CRS-R score and CRS-R subscale scores after HD-tDCS. CRS-R, Coma Recovery Scale-Revised; * *p* < 0.05; ** *p* < 0.01.

After repeated sessions of HD-tDCS, there was a significant increase in total CRS-R score (t = −3.451, *p* = 0.003, Figure 2) and all six subscale scores [auditory (t = −3.230, *p* = 0.004), visual (t = −2.586, *p* = 0.018), motor (t = −2.631, *p* = 0.016), verbal (t = −2.359, *p* = 0.029), communication (t = −2.169, *p* = 0.042), and arousal functions (t = −2.169, *p* = 0.042)].

### 3.2. Changes in EEG Microstate Parameters following HD-tDCS

**Figure 3:**
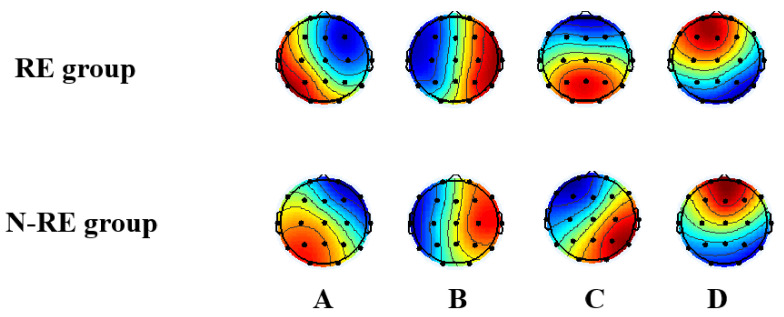
Group mean maps showing the spatial changes in each microstate topology class (A, B, C, D) in responsive and non-responsive groups.

Variance: The maps of the four microstate classes in responsive and non-responsive groups are shown in Figure 3. Among all patients, the four classes of microstate topography explained (75.11 ± 7.07)% of the individual microstate variance before HD-tDCS and (75.13 ± 7.55)% after HD-tDCS.

**Figure 4:**
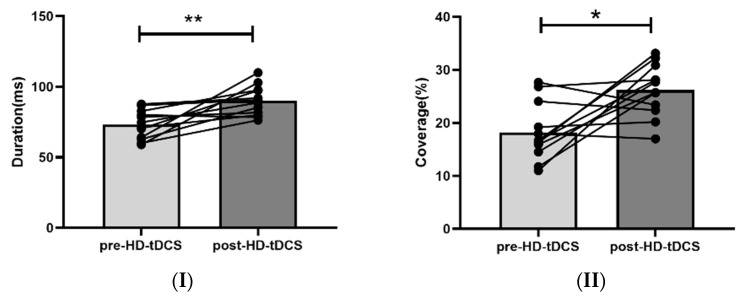
The changes of duration (**I**) and coverage (**II**) of microstate class C parameters before and after HD-tDCS in the responsive group. * *p* < 0.05; ** *p* < 0.01.

Duration: For the RE group, the mean duration of the four microstate classes varied from (89.00 ± 5.85) ms to (92.04 ± 8.72) ms after repeated HD-tDCS. The duration of class C increased significantly from (73.21 ± 10.17) ms at baseline to (90.12 ± 10.43) ms after HD-tDCS (F = 16.153, *p* < 0.05, FDR corrected, Figure 4I), while no significant change in class C duration was measured in the N-RE group ((86.32 ± 25.26) ms to (87.62 ± 22.72) ms, F < 0.001, *p* > 0.05, FDR corrected).

Occurrence: The frequency of individual class C microstates increased significantly from (2.50 ± 0.54)/s to (3.08 ± 0.64)/s following HD-tDCS (F = 5.888, *p* > 0.05, FDR corrected), while the frequency of class D microstates decreased from (3.30 ± 0.76)/s to (2.60 ± 0.82)/s (F = 4.682, *p* > 0.05, FDR corrected), but there is no statistical difference. No significant changes were observed in the N-RE group (class C: F = 0.889, *p* > 0.05, FDR corrected; class D: F = 0.415, *p* > 0.05, FDR corrected).

Coverage: The proportion of time in the class C microstate increased significantly from (18.21 ± 5.38)% at baseline to (27.13 ± 6.90)% after HD-tDCS in the RE group (F = 12.448, *p* < 0.05, FDR corrected Figure 4II), while the proportion of time in the class D microstate decreased from (31.50 ± 12.43)% to (21.96 ± 10.05)% were not significant (F = 4.267, *p* > 0.05, FDR corrected). No changes in coverage were observed in the N-RE group (F = 0.294, *p* > 0.05; F = 1.522, *p* > 0.05, FDR corrected) (Table 2).

**Figure 5:**
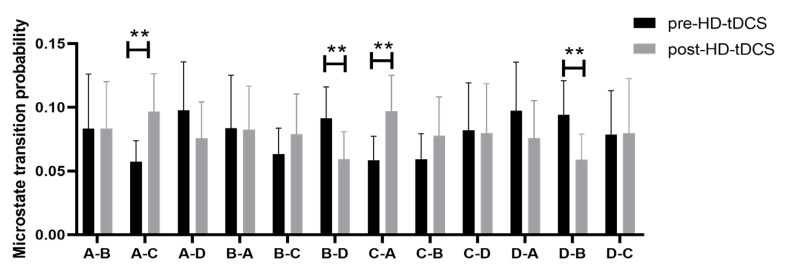
Syntax of microstate class concatenation changes induced by HD-tDCS in the responsive group. (** *p* < 0.01).

Microstate Syntax: The frequencies of both A→C and C→A transitions increased significantly after HD-tDCS, while the frequencies of B→D and D→B transitions significantly decreased (all *p* < 0.05, FDR corrected). There were no changes in transition frequency in the N-RE group (all *p* > 0.05, FDR corrected) (Figure 5).

## 4. Discussion

Resting-state EEG microstates reflect the spatiotemporal activities of large-scale neural networks implicated in arousal and consciousness. Therefore, to identify neural network mechanisms contributing to DOCs and recovery, we analyzed the changes in resting-state EEG microstate profiles of prolonged DOCs patients following HD-tDCS, an intervention shown to promote DOCs recovery in some patients. Indeed, DOCs patients responsive to HD-tDCS (responsive group) demonstrated changes in EEG microstate parameters not observed in non-responsive patients. The most prominent changes were increases in the average duration and coverage of microstate class C, an enhanced probability of microstate A to C transition, and a reduced probability of microstate B and D transition. Collectively, these changes in EEG microstates parameters are associated with the improvement of consciousness.

EEG microstates are proving to be a promising neurophysiological tool for millisecond-scale brain network dynamics in patients with consciousness disorders [14]. The microstates reflect changes in large-scale neural network activity. Koenig and colleagues identified four map configuration clusters of EEG microstates referred to as classes A, B, C, and D. The change in the topographical map of recorded potentials represents a change in the distribution of the underlying active dipoles in the brain that generate the topography. The four resting-state EEG microstate classes (A, B, C, D) explain over 80% of the resting-state data [14,40]. Microstate class A shows a left–right orientation, class B a right–left direction, class C a rostral–caudal direction, and class D a frontal center maximum [41]. In this study, the maps of the four microstate classes in responsive and non-responsive groups are shown in Figure 3. The global explain variance of responsive and non-responsive groups is both over 70%, and it indicated that each EEG sample has a good similarity to the microstate prototype assigned to it [38]. In the responsive group, after tDCS intervention, the changes of microstate C were mainly manifested as the extension of duration, occurrence, and coverage. This study also found an increase in the conversion rate between microstates A and C and a decrease in the conversion rate between B and D. In these studies of EEG microstates, the common time-series parameters mainly include global explained variance, average duration, occurrence, coverage, and microstate syntax [14]. The average duration of a microstate is the average length of time each microstate remains stable while it appears, which is interpreted as reflecting the stability of its underlying neural components. The occurrence of a microstate refers to the average number of times per second a microstate ascends during the recording period. It may reflect a tendency for its underlying neural generator to be activated, while the coverage of microstates is part of the total recording time that microstates dominate. Many studies have shown the relationship between RSNs activity and EEG microstates. Britz et al. showed that microstates A, B, C, and D correspond to RSNs previously associated with phonological processing, the visual network, the salience network, and attention, respectively [16]. Thus, the changes in microstate C in the responsive group may indicate that the salience network also changes after interventions. Due to the lack of relevant imaging evidence in this study, a large amount of fMRI data is still needed to support this inference. However, Guo et al. hold a different view; that is, with the improvement in the CRS-R scores, the microstate C decreases and the microstate D increases [33]. Meanwhile, they believe that the higher level of consciousness corresponds with the decrease in the parameters of microstate C and the increase in the parameters of microstate D. The changes in microstates were still significant after the 2 weeks of HD-tDCS treatment over precuneus. This study showed that the dynamics of EEG brain activity could be modulated by HD-tDCS [33]. Another study reported differences in microstate D frequency between MCS and VS patients and a significant increase in microstate D two weeks after HD-tDCS [42]. These results are different from the results of this study, potentially due to varied stimulation targets, differences in the post-intervention delay in DOCs assessment, and the clinical status of DOCs patients. Thus, microstate parameters may continue to change after post-intervention assessment. Studies with longer serial follow-up evaluations are warranted to examine the long-term neuroplastic changes in network function after HD-tDCS interventions. Microstate analyses are potentially valuable in examining various changes in brain development and behavioral states. The microstate syntax, also known as the probability of transition between microstates, is non-random and usually interpreted as representing the coding sequence activation of the neural components that produce microstates. The microstate transition sequence also has potential significance [39]. Transitions in microstate syntax were also found to be non-random in healthy subjects [39]. In the present study, the number of transitions between microstates A and C increased following tDCS in the responsive group, while transitions between microstates B and D fell following the intervention. Past research has shown that these four microstates reflect the activities of distinct resting-state functional networks [16]. The transition from class A and C may reflect increased connectivity between the auditory or sensorimotor network and the salience network, while connectivity between the visual network and attentional network may decrease in the RE group after HD-tDCS stimulation. Thus, microstate syntax may be a useful biomarker of DOCs and improvement following HC-tDCS. In this study, we analyzed the changes of four classic microstates in the two groups, besides, the changes of five categories and six categories microstates were also explored. See details in Supplementary Materials.

Consciousness is a complex mental process involving multiple interacting neural networks, including DMN, which is functionally related to internal awareness [43] and the frontoparietal network, which processes external stimuli [44]. The DMN is often considered an ‘intrinsic’ system specializing in internally directed cognitive processes such as daydreaming, recall, and future planning [45]. Specific metabolic decreases have been observed in the frontoparietal association cortices of patients with VS and MCS. Patients with MCS maintain partial metabolism in the frontoparietal networks, whereas UWS patients show broad bilateral frontoparietal dysfunction [46]. In addition to the DMN and frontal-executive network, higher-order regions and functional networks such as the salience network [47] have also been implicated in states of reduced or absent consciousness. For instance, a recent neuroimaging study reported that higher-order sensorimotor circuits of the brain’s global network support human consciousness [48].

There is still a certain misdiagnosis rate between MCS and VS based on the clinical behavior scale [1]. Some researchers believe that MCS and VS. can be distinguished based on the coherence changes caused by TDCS in DOCs patients. In this study, we focused on discovering microstate changes induced by TDCS. Repeated HD-tDCS significantly increased the total CRS-R score and the scores of all six CRS-R subscales in the responsive group. Quantifying the differences in EEG microstates between VS and MCS patients before and after treatment could identify possible confounding factors and better draw conclusions about the effectiveness of HD-tDCS treatment. However, because the sample size is small, we will expand the sample size to further analyze changes in more groups of responders in MCS and VS patients in the future.

The present study has several limitations. Firstly, the absence of a sham stimulation group could not explain well that the changes in brain microstates during consciousness recovery were completely caused by HD-tDCS regulation. Additionally, due to the small sample size of this study and the differences in etiology and course of disease among the enrolled patients, it was difficult to reduce the impact of individual patient differences on the study. Third, in this study, it was limiting to only use the CRS-R score as a clinical behavior assessment. Later, we will collect the changes in patients’ clinical signs to help judge the changes in patients’ consciousness disorders. In the future, the rigor of clinical evaluation will be improved, and neuroimaging techniques and larger multi-center studies will be used to better explore the neural mechanisms of consciousness disorders and the recovery of this disease.

## 5. Conclusions

Our study found that HD-tDCS improved the clinical behavioral scores of DOCs patients, and such improvements were observed in EEG microstates. Notably, changes (increases) in class C microstates may indicate that salience network activation is associated with DOCs recovery.

## Figures and Tables

**Table 1:** Demographic information and clinical data of the patients.

ID	Sex	Age	Etiology	Course (Days)	T0 (CRS-R)	T0-Diagnosis	T1 (CRS-R)	T1-Diagnosis	T2 (CRS-R)	T2-Diagnosis	Follow-Up at 3 Months
RE1	M	52	Trauma	84	11	MCS+	11	MCS+	12	MCS+	EMCS
RE2	F	49	HIE	30	6	MCS-	7	MCS-	15	MCS+	EMCS
RE3	M	53	Trauma	34	5	MCS-	7	MCS-	14	MCS+	EMCS
RE4	F	74	Hemorrhage	101	11	MCS+	11	MCS+	15	MCS+	Dead
RE5	M	49	Hemorrhage	50	5	VS	6	VS	7	VS	VS
RE6	M	55	Trauma	302	6	VS	6	VS	8	MCS-	MCS-
RE7	M	72	CI	42	5	MCS-	5	MCS-	9	MCS+	MCS+
RE8	M	47	Hemorrhage	29	6	VS	6	VS	12	MCS+	MCS+
RE9	M	58	Trauma	53	9	MCS-	9	MCS-	10	MCS-	EMCS
RE10	F	68	Hemorrhage	30	8	MCS-	9	MCS+	15	MCS+	MCS+
RE11	M	59	CI	68	5	VS	5	VS	6	MCS+	MCS+
RE12	M	72	CI	200	2	VS	2	VS	4	VS	VS
N-RE1	M	54	Hemorrhage	73	6	VS	6	VS	6	VS	VS
N-RE2	M	56	HIE	41	2	VS	2	VS	2	VS	VS
N-RE3	F	39	HIE	128	4	VS	4	VS	4	VS	VS
N-RE4	M	18	Disseminated encephalomyelitis	48	4	VS	4	VS	4	VS	MCS-
N-RE5	M	56	Hemorrhage	88	3	VS	3	VS	3	VS	VS
N-RE6	M	64	Hemorrhage	34	10	MCS-	10	MCS-	10	MCS-	MCS-
N-RE7	F	70	CI	58	4	VS	4	VS	4	VS	MCS+
N-RE8	F	39	HIE	215	3	VS	3	VS	3	VS	VS
N-RE9	M	57	Hemorrhage	52	6	VS	6	VS	6	VS	MCS+

T0, time before HD-tDCS; T1, after a single session of HD-tDCS; T2, after 10 sessions of HD-tDCS for 5 days; RE, responsive group; N-RE, non-responsive group; HIE, hypoxic-ischemic encephalopathy; CI, cerebral infarction; CRS-R, Coma Recovery Scale-Revised; MCS, minimally conscious states; VS, vegetative state; EMCS, emergence from minimally conscious.

**Table 2:** Microstate parameter changes following HD-tDCS in responsive and non-responsive patients.

	RE Group			N-RE Group		
	Before HD-tDCS	After HD-tDCS	*p* Value	Before HD-tDCS	After HD-tDCS	*p* Value
**Microstate class A**						
Duration (ms)	85.37 ± 14.36	91.04 ± 13.05	0.323	84.34 ± 25.71	87.23 ± 33.13	0.839
Occurrence (per s)	2.97 ± 0.65	3.09 ± 0.51	0.618	3.10 ± 1.06	3.16 ± 1.18	0.91
Coverage (%)	25.26 ± 7.87	27.32 ± 5.77	0.473	23.63 ± 5.66	25.44 ± 8.51	0.603
**Microstate class B**						
Duration (ms)	84.74 ± 15.39	87.99 ± 20.50	0.665	87.57 ± 22.47	82.08 ± 23.23	0.617
Occurrence (per s)	2.94 ± 0.61	2.64 ± 0.74	0.297	3.55 ± 1.65	3.10 ± 1.47	0.555
Coverage (%)	25.03 ± 8.62	23.61 ± 10.54	0.722	27.83 ± 6.54	23.30 ± 7.28	0.184
**Microstate class C**						
Duration (ms)	73.21 ± 10.17	90.12 ± 10.43	0.001 *	85.41 ± 25.93	85.38 ± 26.29	0.999
Occurrence (per s)	2.50 ± 0.54	3.08 ± 0.64	0.024	3.38 ± 1.14	2.96 ± 0.67	0.36
Coverage (%)	18.21 ± 5.38	27.13 ± 6.90	0.002 *	26.03 ± 5.851	24.33 ± 7.413	0.595
**Microstate class D**						
Duration (ms)	95.05 ± 21.41	84.21 ± 19.47	0.208	80.18 ± 31.20	83.52 ± 20.59	0.793
Occurrence (per s)	3.30 ± 0.76	2.60 ± 0.82	0.042	3.11 ± 1.14	3.53 ± 1.58	0.528
Coverage (%)	31.50 ± 12.43	21.96 ± 10.05	0.051	22.56 ± 6.65	26.92 ± 8.27	0.235

*, *p* < 0.05.

## Data Availability

The data that support the findings of this study are available from the corresponding author upon reasonable request.

## Supplementary Materials

The following are available online at https://www.mdpi.com/article/10.3390/brainsci13010005/s1, Figure S1: Group mean maps showing the spatial changes in each microstate topology class in responsive group (5 categories and 6 categories), Figure S2: (1) Duration changes before and after high definition transcranial direct current stimulation intervention of microstate B of 5 categories in responsive group; (2) Duration changes before and after high definition transcranial direct current stimulation intervention of microstate B of 6 categories in responsive group; Table S1: Microstate parameter changes of 5 categories following HD-tDCS in responsive and non-responsive patients, Table S2: Microstate parameter changes of 6 categories following HD-tDCS in responsive and non-responsive patients.
