# Supplementary material for: Dynamic Changes of Brain Activity in Different Responsive Groups of Patients with Prolonged Disorders of Consciousness
Source: Brain Sci. 2022 Dec 20;13(1):5. doi: 10.3390/brainsci13010005 (PMC9856292; doi:10.3390/brainsci13010005)
Supplement: Supplementary file 1 [file brainsci-13-00005-s001.zip › brainsci-2022020-supplementary.pdf]

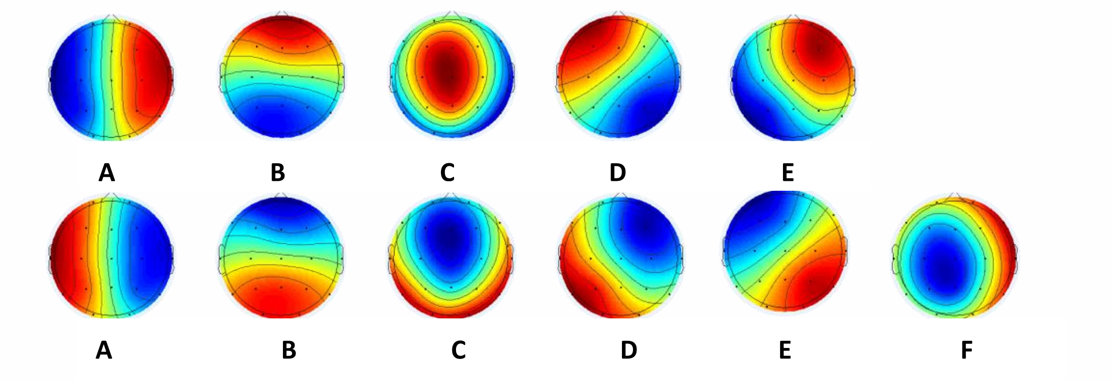

Figure S1. Group mean maps showing the spatial changes in each microstate topology class in responsive group (5 categories and 6 categories)

Discussion: Regardless of the 5 categories or 6 categories microstate analysis, the difference in microstate parameters before and after HD-tDCS intervention was found to be significant in responsive group, and was only found in microstate B.

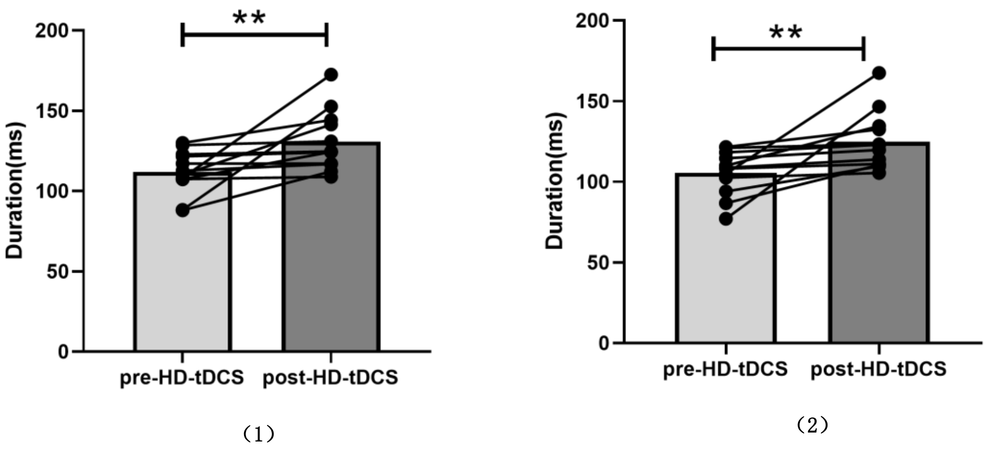

Figure S2. (1) Duration changes before and after high definition transcranial direct current stimulation intervention of microstate B of 5 categories in responsive group; (2) Duration changes before and after high definition transcranial direct current stimulation intervention of microstate B of 6 categories in responsive group. ***p* < 0.01.

Table S1. Microstate parameter changes of 5 categories following HD-tDCS in responsive and non-responsive patients.

	responsive group			non-responsive group		
	before-HD-tDCS	after-HD-tDCS	*p* value	before-HD-tDCS	after-HD-tDCS	*p* value
**Microstate classes A**						
Duration(ms)	109.34±24.61	106.48±22.16	0.768	157.48±55.75	150.21±49.28	0.773
Occurrence(per sec)	7.41±1.57	6.68±1.66	0.285	9.26±4.11	8.30±3.00	0.579
Coverage(%)	20.93±8.49	18.55±7.49	0.493	33.37±7.28	29.20±6.65	0.222
**Microstate classes B**						
Duration(ms)	111.89±13.57	130.87±18.67	*0.009**	76.835±17.44	81.21±17.75	0.604
Occurrence(per sec)	7.76±1.57	8.37±1.57	0.355	1.86±0.71	3.48±2.26	0.506
Coverage(%)	21.59±5.25	28.04±7.92	0.028	3.28±3.34	8.12±5.67	0.415
**Microstate classes C**						
Duration(ms)	113.76±23.14	105.88±18.63	0.368	117.09±30.65	119.17±134.39	0.849
Occurrence(per sec)	6.60±2.87	5.04±2.30	0.156	4.81±1.87	5.09±0.96	0.852
Coverage(%)	18.83±10.44	13.34±8.27	0.167	14.05±6.06	14.51±3.64	0.693
**Microstate classes D**						
Duration(ms)	106.72±11.99	115.13±19.65	0.219	165.83±52.95	160.92±55.85	0.852
Occurrence(per sec)	7.24±0.83	7.47±1.31	0.614	8.18±2.39	7.63±3.80	0.457
Coverage(%)	19.00±2.63	21.77±6.11	0.163	34.67±7.44	31.87±11.9	0.56
**Microstate classes E**						
Duration(ms)	106.28±11.65	105.58±13.95	0.891	103.63±15.69	111.29±15.69	0.455
Occurrence(per sec)	7.50±0.98	6.94±1.02	0.163	5.83±3.76	6.20±3.70	0.84
Coverage(%)	19.72±3.10	18.28±4.02	0.336	14.60±9.17	16.28±7.38	0.674

*, $p < 0.05$

Table S2. Microstate parameter changes of 6 categories following HD-tDCS in responsive and non-responsive patients.

	responsive group			non-responsive group		
	before-HD-tDCS	after-HD-tDCS	*p* value	before-HD-tDCS	after-HD-tDCS	*p* value
**Microstate classes A**						
Duration(ms)	106.30±24.43	103.53±21.05	0.769	121.83±36.98	123.97±37.75	0.905
Occurrence(per sec)	6.91±1.52	6.21±1.60	0.286	5.66±1.46	5.75±1.95	0.911
Coverage(%)	18.87±7.77	16.69±6.75	0.47	17.46±7.31	18.11±7.06	0.851
**Microstate classes B**						
Duration(ms)	105.66±13.72	124.81±18.08	*0.008**	77.27±12.52	73.55±10.74	0.509
Occurrence(per sec)	6.66±1.49	7.50±1.54	0.189	1.70±0.69	2.14±1.37	0.779
Coverage(%)	17.51±4.80	24.10±8.01	0.023	3.03±1.17	3.82±2.55	0.782
**Microstate classes C**						
Duration(ms)	103.89±18.93	98.05±16.46	0.429	95.74±17.53	98.35±20.38	0.774
Occurrence(per sec)	6.19±2.80	4.93±2.39	0.258	4.05±2.69	4.98±1.76	0.686
Coverage(%)	16.29±9.30	12.20±7.89	0.249	19.14±3.50	19.67±4.07	0.666
**Microstate classes D**						
Duration(ms)	102.46±12.73	102.94±15.12	0.934	168.90±61.63	163.20±54.27	0.838
Occurrence(per sec)	6.78±1.02	6.32±1.02	0.289	9.64±5.30	8.54±3.98	0.626
Coverage(%)	17.71±3.44	16.74±3.68	0.566	36.87±11.10	32.67±8.26	0.376
**Microstate classes E**						
Duration(ms)	103.84±11.73	112.59±19.58	0.198	77.15±18.33	81.17±18.05	0.646
Occurrence(per sec)	6.91±0.79	7.13±1.25	0.61	1.93±0.67	3.21±1.94	0.542
Coverage(%)	17.69±2.72	20.37±5.94	0.169	3.51±1.12	7.25±4.69	0.45
**Microstate classes F**						
Duration(ms)	96.71±15.30	95.00±15.45	0.788	152.74±45.62	150.85±49.84	0.934
Occurrence(per sec)	5.25±1.60	4.44±1.44	0.207	8.24±2.24	7.20±3.71	0.483
Coverage(%)	12.43±4.50	10.32±4.02	0.239	29.86±5.67	28.03±10.42	0.649

*, $p < 0.05$.
